# The need for and acceptability of a cancer training course for medical and nursing students in Tanzania: a convergent mixed methods study

**DOI:** 10.1186/s12909-024-05497-w

**Published:** 2024-06-03

**Authors:** Stella Emmanuel Mushy, Dickson A. Mkoka, Gift G. Lukumay, Agnes F. Massae, Corissa T. Rohloff, Lucy R. Mgopa, Dorkasi L. Mwakawanga, Nidhi Kohli, Michael W. Ross, Ever Mkonyi, Maria Trent, Kibwanda Athumani, Shalini Kulasingam, B. R. Simon Rosser

**Affiliations:** 1 School of Nursing Department of Community Health Nursing, Muhimbili University of Health and Allied Sciences, Dar es Salaam, Tanzania; 2 Department of Educational Psychology, University of Minnesota, Minneapolis, MN USA; 3 Department of Psychiatric and Mental Health, School of Medicine, Muhimbili University of Health and Allied Sciences, Dar es Salaam, Tanzania; 4 Institute for Sexual and Gender Health, Department of Family Medicine and Community Health, University of Minnesota Medical School, Minneapolis, MN USA; 5 Division of Epidemiology and Community Health, School of Public Health, University of Minnesota, Minneapolis, MN USA; 6 Schools of Medicine, Nursing, and Public Health, Johns Hopkins University, Baltimore, Maryland, US; 7 School of Nursing and Midwifery, The Muhimbili University of Health and Allied Sciences, Dar es Salaam, 65004 Tanzania

**Keywords:** Cancer, Nursing students, Medical students, Education, Training, Cancer care, Need and acceptability, Africa, Tanzania

## Abstract

**Background:**

The cancer burden in Africa is on the rise. A Cancer Training Course on screening, prevention, care, and community education is crucial for addressing a wide range of cancer health issues. When appropriately educated healthcare providers on cancer provide care, patient care improves, and healthcare costs decrease. However, in Tanzania, doctors and nurses receive little or no training in primary cancer care in their bachelor’s program.

**Aim:**

This study assessed the need and acceptability of a cancer training course for nursing and medical doctor students at the Muhimbili University of Health and Allied Sciences (MUHAS) in Dar es Salaam, Tanzania.

**Methods:**

This study was a cross-sectional parallel mixed method study during the 3-month follow-up within the larger study on sexual health training for health professionals. The study was a randomized controlled (RCT), single-blind, parallel trial of sexual health training versus a waitlist control among health students at MUHAS in Tanzania. Descriptive analysis was performed to analyze the participants’ demographic information, need, and acceptability of the cancer training courseto determine the frequencies and percentages of their distribution between disciplines. In addition, inductive thematic analysis was performed for the qualitative data. The RCT study was registered at Clinical Trial.gov (NCT03923582; 01/05/2021).

**Results:**

Data were collected from 408 students (272 medical doctors and 136 nursing students). The median age of the participants was 23 years. Most (86.0%) medical and 78.1% of nursing students reported receiving little to no cancer training. On the other hand, most (92.3%) medical and nursing (92.0%) students were interested in receiving cancer training. Furthermore, 94.1% of medical and 92.0% of nursing students needed a cancer training course in their undergraduate program. In addition, participants said a cancer training course would be important because it would help them improve the quality of cancer care and enhance the quality of life for patients by ensuring early diagnosis and treatment.

**Conclusion:**

A cancer training course is both highly needed and acceptable to medical and nursing students. Implementation of this cancer training course will improve students’ knowledge and skills and eventually improve the quality of cancer care and patients’ quality of life by ensuring early diagnosis and management.

## Background

Cancer is an emerging crisis and a health concern in Africa [1, 2]. Cancer was the second leading cause of death globally in 2020 [2], with a majority (70%) of deaths occurring in developing African countries [3]. Cancer incidence in Africa is expected to reach 1.28 million cases and 970,000 deaths annually by 2030 [3], with breast cancer as the leading cause of cancer death in African women [4].

Cancer prevention has not been a priority in Africa and was once the least prioritized health concern in Sub-Saharan Africa [5], having previously been thought to be only a Western public health problem and concern. However, as the population ages and embraces globalized lifestyle changes, cancer is no longer a health problem limited to the West [5]. People in African countries suffer significantly from cancer but have few resources, including personnel and facilities, to address this in a timely manner and are frequently diagnosed too late [6]. More than 1.27 million people in Africa are predicted to die from cancer annually by 2030 if no measures are taken to address the growing burden of cancer [6].

East Africa, including Tanzania, has Africa’s second-highest cancer rate [2]. Cancer-related deaths in Tanzanian hospitals were higher (56.5%) among those aged 15 to 59 years than among those aged *≤* 15 years and *≥* 60 years, and females outnumbered males [7]. Cancers of the breast, cervix, esophagus, and liver were the leading causes of death, with females dying mostly from cancers of the cervix, esophagus, and liver and males dying mostly from cancers of the esophagus, liver, and prostate [7]. Regional differences in cancer types also exist. Cancers of the liver and lymphoma predominate in the Lake Victoria zone, while Kaposi’s sarcoma is more common in the southern highlands and southwest zones [7]. The incidence of breast cancer in Tanzania is projected to increase 82% by 2030 from 2017 [8].

Currently, 80% of Africans with cancer present with advanced disease [5]. Top cancer risk factors in Tanzania include high rates of human papillomavirus (HPV), human immunodeficiency virus (HIV), smoking, alcohol, and obesity [7]. Therefore, cancer training course must be prioritized in Tanzania to reduce cancer incidence. Doctors and nurses are the health team’s core members, providing most healthcare services [9]. Doctors and nurses are respected and valued in Tanzania as community health experts who advise at all community levels, within government, and in policy development. What health professionals (doctors, nurses, and midwives) say, believe, and advocate for in terms of primary cancer care significantly impacts local communities [1]. Skilled nurses and midwives are critical multidisciplinary healthcare team members because they ensure that patients receive quality care through evidence-based nursing care for prevention, early detection, and treatment [1].

Cancer training course of health providers is critical for addressing various cancer health issues. When nurses and midwives are appropriately educated, patient care improves, and healthcare costs decrease [1]. Unfortunately, only eight African countries offer cancer training courses in their undergraduate program, and Tanzania is not on the list [1]. In Tanzania, doctors and nurses receive little or no training in primary cancer care in their respective training programs.

The National Institutes of Health funded a five-year (2018–2023) randomized controlled trial (RCT) called the “Training for Health Professionals (THP)” study. The primary aim of this project was to evaluate a comprehensive, Afrocentric sexual health training curriculum for health students at the Muhimbili University of Health and Allied Sciences (MUHAS) in Dar es Salaam. At the 3-month follow-up assessment, we assessed the need for and acceptability of cancer training course. In this course, cancer training encompasses education on cancer screening, prevention, care, and community education.

## Methods

### Study design and setting

The need for and acceptability data were assessed cross-sectionally during the 3-month follow-up in December 2021 within the larger THP study. This study was an RCT, single-blind, parallel trial of an Afrocentric comprehensive sexual health training versus a waitlist control among health students at MUHAS in Dar es Salaam, Tanzania. Details regarding this RCT have been published elsewhere [10]. The RCT study was registered at ClinicalTrials.gov (NCT03923582; 01/05/2021).

### Study population and sample size

Nursing students in years 2 and 3 and medical students in years 3 and 4 were eligible for participation based on the fact that they had sufficient coursework to provide the background needed to answer the research questions. Need and acceptability data were collected from 408 students (271 medical and 137 nursing students) (Table 1).

### Study procedures

At the 3-month follow-up, the students completed a final skills assessment that concluded with an online Qualtrics survey asking them to reflect on their experiences in the study. The survey took approximately 15 min and included reflections on their performance on the skills assessment, their evaluation of the study activities, and the need and acceptability of cancer training course for medical and nursing students. In addition, five questions were asked on the need for and acceptability of the cancer training course, including the following: (1) How much training on cancer have you received? (2) How interested are you in receiving cancer training? (3) How would you like to receive the training? (4) Is it acceptable for them to receive the training, and (5) What could be the value or importance of the training? The first four questions were closed-ended items. The fifth question was an open-ended, qualitative item where the students could write their thoughts on the value or importance of receiving cancer training. All the survey responses were automatically saved in the Qualtrics online database. Extracted data from Qualtrics were deidentified and then kept in secure storage with password protection, with a few staff accessing the files.

### Data analysis

#### Quantitative data

Data were descriptively analyzed using IBM SPSS Statistics version 24.0. Descriptive statistics were used to summarize participants’ demographic information, need for, and acceptability of the cancer training course. .

### Qualitative data

The survey asked questions in English; however, participants were free to answer in either English (the language of instruction) or Kiswahili (the lingua franca in Tanzania). No translation was required for the open-ended item, as all the answers were written in English.

All the responses from open-ended questions were extracted, coded, and analyzed thematically.

Two coresearchers (SEM & AFM) used line-by-line open coding for all responses, discussed the contents, and agreed to the interpretations. To ensure intercoder reliability, the two coders discussed discrepancies between their codes until they reached agreement. After defining the codes, the first author coded all parts of the transcript, and the fourth author reviewed the codes. We inductively created a general list of codes from the responses, examined similarities and differences, and grouped them into subthemes. The focus was on broad patterns in the data, and the coded data were combined based on their relationships to form subthemes and themes. Finally, the identified themes were reviewed and discussed with the team of researchers.

## Results

### Sociodemographic characteristics of the participants

Details on the sociodemographic information of the participants are shown in Table 1. The median age of the participants was 23 years (IR 2.46) [age range, 21–38 years]. Most participants (66.4%) were medical students, and half (51.0%) were in their penultimate year of study at MUHAS. 67% of the participants were male, the majority (92.7%) were single, and most (85.3%) were Christian.

**Table 1:** Sociodemographic characteristics of the study participants

	Frequency (*n*)	Proportion (%)
**Age (M, IR)**	23 (2.46)	
**Discipline**		
Nursing Medicine	137 271	33.6 **66.4**
**Current year in training at MUHAS**		
Penultimate Final	208 200	**51.0** 49.0
**Gender**		
Male Female Other/Preferred not to answer	275 123 10	**67.4** 30.2 2.4
**Relationship status**		
Single Cohabiting Married Other	378 8 17 5	**92.7** 1.9 4.2 1.2
**Religion**		
Christian Muslim Other/Preferred Not to Answer	348 56 4	**85.3** 13.7 1.1

### Need for and acceptability of the cancer training course

#### Quantitative results

The need for and acceptability of the cancer training course for nursing and medical students are shown in Table 2. The results showed that 86.0% of medical and 78.1% of nursing students had received little to no cancer training. Most medical (92.3%) and nursing (92.0%) students showed interest in receiving more cancer training. Almost all medical (94.1%) and nursing (92.0%) students reported that it would be acceptable for them to receive cancer training course during their undergraduate program. In addition, 63.5% of medical and 53.3% of nursing students preferred to receive cancer training course as a week-long course during the school holiday. The response rates for multiple item-response categories were low, including categories with zero responses (e.g., no one indicated they were uninterested or very uninterested in receiving more cancer training), so we did not conduct statistical tests comparing medical and nursing students for these items.

**Table 2:** Need and acceptability of cancer training course for nursing and medical students

Items	Medical students		Nursing students	
	Frequency (*n*)	Proportion (%)	Frequency (*n*)	Proportion (%)
How much training have you received in cancer screening, prevention, care, and community education?				
Well educated A lot A little None	17 21 188 45	6.3 7.8 **69.4** 16.6	16 14 76 31	11.7 10.2 **55.5** 22.6
**Would you be interested in receiving more cancer screening, prevention, care, and community education training?**				
Very interested Interested Neutral Uninterested Very uninterested	250 19 2 0 0	**92.3** 7.0 0.7 0.0 0.0	126 11 0 0 0	**92.0** 8.03 0.00 0.00 0.00
**It would be acceptable for me to attend training in cancer screening, prevention, care, and community education**				
Strongly agree Agree Neither agree nor disagree Disagree Strongly disagree	255 16 0 0 0	**94.1** 5.9 0.0 0.0 0.0	126 9 1 1 0	**92.0** 6.6 0.7 0.7 0.0
**How would you like to receive this cancer training?** ***“Choose all that apply.”***				
As an evening class for 2–3 h per week throughout the semester As a week-long course during the holiday As an online course Others	95 172 62 11	35.1 **63.5** 22.9 4.1	54 73 30 11	39.4 **53.3** 21.9 8.0

### Qualitative results

Participants were asked to indicate the importance of cancer curriculum training. Two major themes emerged from the analysis: improvement of quality of cancer care and quality of life (QOL) for patients diagnosed with cancer.

1. Improves the quality of cancer care offered.

Participants mention that upon receiving cancer training course, they can confidently provide quality care to patients because the training equips them with the necessary knowledge and skills related to cancer management, enabling them to diagnose and treat cancer at an early stage

*“Having skills in cancer management helps us to recognize the persons at risk, identify the patients at an early stage, and allows early treatment.”****Nursing student, third year***.

Additionally, possessing cancer care skills will empower the participants to raise awareness in the community about the risk factors for cancer. Ultimately, this ability will encourage community members to visit health facilities for the screening of various cancer types.

*“Cancer training will improve our community cancer care skills, enabling us to raise awareness within the community about the importance of attending nearby health facilities for cancer screening to identify individuals at risk and provide early treatment whenever possible.”****Nursing student, third year***.

Enhances the quality of life for patients diagnosed with cancer.

Participants expressed that receiving cancer training would equip them to deliver quality cancer services to patients within both health facilities and the community at the earliest opportunity.

This, they believe, will contribute to improving the quality of life for patients with cancer, particularly when they receive treatment during the initial stages of the disease.

*“If the community receives cancer screening services from skilled healthcare providers, it will help diagnose cancer cases at early stages and ensure early management, eventually improving quality of their life.”****Medical doctoral student, fourth year***.

Participants further said, acquiring skills on community education for cancer would, in their view, improve cancer awareness in the community through sensitization. This, in turn, is expected to enhance the uptake of cancer services and health-seeking behaviors.

*“Educating the community about cancer will raise awareness and knowledge*, empowering individuals to modify risky *behaviours or seek early care and treatment.”****Nursing student, third year***.

It was noted that most people in the community lack sufficient knowledge about cancer and predominantly relies on traditional practices. This often results in delayed medical treatment, with individuals seeking medical assistance only when the disease has already reached an advanced stage

*“Most people lack knowledge about cancer, and most have traditional practices, leading to delays in seeking medical treatment.”****Medical doctoral student, fourth year***.

Participants reported that a cancer training course will provide clinicians with the skills to advocate for regarding early cancer detection behavior uptake and treatment, ultimately improving the quality of life for individuals diagnosed and treated for cancer at its earliest stage.

*“I believe that this cancer training course will be of great significance, as it will raise awareness among clinicians who can advocate for a better understanding within society regarding the early detection and treatment of cervical cancer.****“Medical student, fourth year.***

## Discussion

This study assessed the need for and acceptability of cancer training course for Tanzania’s nursing and medical doctor students. The high demand for and acceptability of a cancer training course among Tanzanian medical and nursing students is particularly important given the resource constraints of the national health system. In Tanzania, access to specialty care is limited, and even patients with access to oncological services have been shown to present for care at an advanced disease stage [11, 12]. Low awareness of cancer symptoms, long travel time to specialty facilities, and fear of medical treatments all inhibit Tanzanian patients from seeking cancer care at early stages [13]. As a result, most cancer diagnoses in Tanzania are made at late stages [14], which is associated with poorer treatment outcomes and increased mortality. However, late diagnoses cannot be attributed solely to delayed presentation by patients. Weak and confusing referral systems, insufficient pathology and laboratory capacity at health facilities, and high out-of-pocket costs are all contributing factors to late diagnoses [15]. While nurses and physicians in Tanzania must contend with the structural barriers that exacerbate poor cancer outcomes, a more highly trained primary care workforce may improve these outcomes by minimizing the need for referrals, and the associated time and financial burdens.

The inadequate training of primary healthcare providers in the diagnosis and treatment of cancer, particularly in low- and middle-income countries, has been documented throughout the world [16–19]. However, this issue can be improved, as other countries have demonstrated the population-level benefit of including training in cancer care in medical and nursing education. In Malaysia, the training of healthcare providers in cancer care has been shown to significantly contribute to the downstaging of cancers, thereby leading to improved survival rates [20]. Training healthcare providers in the early detection of cancer symptoms, combined with other interventions such as increased public awareness campaigns [21, 22], holds the potential to enhance cancer outcomes in the East African region. This training may cultivate a heightened awareness of risk factors, symptoms, and treatment modalities for various cancer sites, potentially resulting in the prompt detection of cancer symptoms, early referral, and timely treatment, respectively.

Effective cancer management is achieved when a multidisciplinary team and well-skilled providers play a central role in a functioning team [1]. The goal of reducing cancer incidence, increasing survival rate, providing better palliative care, and improving the quality of the patient’s life cannot be realized without having well-trained healthcare providers. A healthcare provider with cancer knowledge and management skills can meaningfully contribute to early detection programs, provide early treatments, and anticipate complications [23]. Healthcare providers with cancer care competency can carry out cancer control activities in different settings, including hospitals, communities, homes, and palliative care units [24]. Nurses with cancer skills are crucial in cancer treatment decision-making [25] and can deliver more complex and advanced care to seriously ill patients [26]. In Tanzania, nursing oncology education as a specialty is not yet well developed. Most nurses in Tanzania learn how to care for individuals with cancer through in-service training in their health facility institutions. With the fast-growing rate of the population with cancer in Tanzania, the country will benefit from having health professionals, especially nurses, with cancer skills early in their careers. Therefore, cancer awareness, prevention, screening, and early intervention must be prioritized in Tanzania to reduce cancer morbidity and mortality.

### Strengths and limitations

Based on a review of the literature, this is the first study to assess the need for and acceptability of cancer training course for undergraduate medical and nursing students in Tanzania. This study informs future studies in developing a culturally acceptable cancer training course tailored to the African context, specifically Tanzania, since most participants showed the need and interest in incorporating a cancer training course into their undergraduate programs. Another key strength of this study includes the substantial sample size, comparison of medical and nursing students, and the siting of the study in Tanzania.

Limitation of this study is that, as the first cancer training course needs and acceptability assessment in Tanzania, we cannot know the reliability of these findings. Second, the findings may not generalize beyond MUHAS medical and nursing undergraduate students to other universities in Tanzania.

## Conclusion

The findings from the study confirm that cancer training course in undergraduate programs is needed and highly acceptable. Most participants stated that they are interested in receiving cancer training course to improve their knowledge and skills to provide quality cancer care. Therefore, implementing cancer training will increase students’ knowledge and skills and eventually improves the quality of life for patients diagnosed with cancer by ensuring early diagnosis and management.

## Data Availability

The datasets generated and/or analyzed during the current study are not publicly available because the participants did not provide their approval for sharing their information. However, data are available from the corresponding author upon reasonable request.

## Abbreviations

HIV: Human Immunodeficiency Virus
MUHAS: Muhimbili University of Health and Allied Sciences
QOL: Quality Of Life
RCT: Randomized Clinical Trial
THP: Training for Health Professionals
